# On Wounds of the Stomach

**Published:** 1817-11

**Authors:** 


*For the London Medical and Physical Journal.*

### *On Wounds of the Stomach.*

BARON PERCY read, at a recent Sitting of the Faculty of Medicine, a memoir on a Wound of the Stomach, with Hernia, occasioned by the patient, a boy of twelve years of age, falling from a tree upon a stake in a hedge:—the aliments he had taken escaped by the wound, and were also returned to the mouth by violent vomiting. The intestines were returned, and the wound sewed up; and, with great attention, the boy was cured. The memoir which we thus abstract was accompanied with an important commentary by the learned Professor, which we shall give at length.

In order that the stomach should be wounded, it required that it should be in a state of repletion, which is generally the case in wounds of this nature which happen after orgies, or in a state of intoxication; but in this case they are nearly always mortal, from the effusion of alimentary matters in the abdominal cavity; at least, unless a happy chance similar to that which saved the life of this youth,—the viscus present itself immediately at the external wound, and is thus evacuated. In twenty sword, bayonet, and knife wounds, with lesion of the stomach, I do not recollect having seen above four or five recover. Our André Paré found the chances of recovery still more rare; therefore, he recommends not to touch these wounds, which he considered mortal, except Nature, as she sometimes does, worked a miracle.

This was the opinion of the surgeons of his time, and the tradition was deduced from the Arabs, though they made an exception, which Paré forgot to make. It is that, when the external wound is large, that of the stomach is to be sewed up. *Ventriculi verò vulnus naturæ demittatur, et si amplum fuerit, si potest, ut de intestinis dictum est, conseratur.* This shews us that the most ancient surgeons practised the suture of the intestines, and it is proved that they knew the method

1 attributed attributed to Rhamdor. André de la Croix, from whom I have borrowed the above passage, insists, as a condition, on the size of the wound in the teguments and muscles; but he does not advise the establishing it if it do not exist, and few authors have thought of this plan, which, at the present day, would present no difficulties to an experienced surgeon. The experiments made by the late M. le Gallois, and myself (who simply served him as assistant), on dogs, whose stomachs we opened and sewed up, and cured in a very little time—those which Messieurs Majendie, Marjolin, and Beclard, have also made with the same result—support our opinion; in favour of which, indeed, I could cite a great number of facts. I will commence with one of the most recent:—M. Richstrat, having had to treat a workman who had received a wound in the epigastric region, judged, from the substance which escaped by the wound, and at the same time by the mouth, that the stomach was wounded; and, as the lesion corresponded with that of the parietes of the abdomen, which was above two inches long, he drew out for a moment the ventricle, and made on it five points of suture. The patient was cured; but M. Kluyskein, of Ghent, who briefly relates this case in the Annals of Foreign Medical Literature, vol. ii. page 289, does not specify the species of suture he made, which it would be extremely curious and important to know, for it is a very different thing to use the suture called that of Pelletier, absurdly recommended by the greater part of medical works, and that with loops. It is the latter that I preferred the only time that I had to make a suture of the stomach. It was in a drummer of the 109th demi-brigade belonging to the corps commanded by General Lecourbe, and during the Swiss war. The drummer, having the fever, and being scarcely able to crawl along, had remained behind, when we retreated before Suwarrow. Some Piedmontese troops of the vanguard of the Russian army came up with him, and, in the most cowardly manner, stabbed him in the body five times with their sabres. They were all in the abdomen: the one situated near the left hypochondrium was four fingers long, and had opened the stomach. Sour milk, with which this unfortunate man had slaked his thirst an hour before, issued from the wound. The enemy's vanguard being repulsed, our brave surgeons brought off the drummer. He was in a dying state. Every effort that he made to vomit, the stomach presented itself at the wound, with its division, by which clotted milk still continued to exude. The surgeon-major Briot, and myself, determined to draw it out with our fingers and dissecting forceps, and to make a continued, but very loose, suture; in the loops of which we placed a

 pencil pencil one of our assistants lent us, and of which each end rested on the teguments beyond the points of union of the external wound: by this means, the stomach could neither withdraw or conceal internally its wound, and we had the power of closing the suture at pleasure. The fate of our army becoming every day more uncertain, the drummer was removed with great precaution, first to Zurich, where he was attended by the Surgeon-major Willaume, and afterwards to Koningsfelden, where my colleague Bacon attended him, and had the satisfaction to see him cured. The threads were cut and withdrawn the twenty-eighth day.

It is not known what kind of suture was made by the brothers Schenkel on the inhabitant of Fulda, who had the stomach opened by the wound of a hunting-knife, and which they were fortunate enough to cure. Nor are we acquainted with that made by Floriano Mathis on the peasant, from the stomach of whom he extracted, by incision, a knife nine inches long, which had been swallowed for a wager some months before. Three examples of *cultrivores* (knife-swallowers,—*Trans.*) on whom the operation was successfully made, my honourable colleagues Des Genettes and Larrey saw, as well as myself, in the Library of Koningsberg, the knife which had been extracted from the stomach of one of them. The surgeon who operated on William Clark in 1699, did not fail to stitch up the stomach, leaving the ends of the thread outside the wound: this was acting with prudence and method, but of what kind the suture was we do not know. It is, however, far from being an indifferent matter. The suture of Pelletier is to be proscribed in this case, although several very respectable practitioners advise it. If it be easy to make, when, across a large wound, the stomach is accessible to the fingers and the needle: it is extremely difficult to undo when the cicatrix is nearly terminated: it is, besides, subject to tear in a multitude of points, which have also the inconvenience of increasing the irritation of the organ. I say the irritation, for we cannot refuse to the stomach a considerable degree of irritability; but it is contractile, as the author of the note asserts, who says that when he handled it he felt it contract and expand alternately; but which, after all, could only extend to a contractility of the tissue, and does not invalidate the result of the experiments of which M. Majendie has rendered us witnesses.

The author here makes some observations on the obscurity of the memoir, and then adds, To conclude, the object of the suture, be it of what kind it may, is not immediately to unite the lips of the wound of the gastric members, but but to render oneself master of this union, and to prevent, as we have already observed, and of which no one can be ignorant, the effusion of the aliments, beverage, blood, and pus in the abdominal cavity. It may be proper to observe, that divided membranes do not heal between themselves: it is by their adherence to the parts with which we hold them in contact that their wounds are cured.

